# Cell migration through three-dimensional confining pores: speed accelerations by deformation and recoil of the nucleus

**DOI:** 10.1098/rstb.2018.0225

**Published:** 2019-07-01

**Authors:** Marina Krause, Feng Wei Yang, Mariska te Lindert, Philipp Isermann, Jan Schepens, Ralph J. A. Maas, Chandrasekhar Venkataraman, Jan Lammerding, Anotida Madzvamuse, Wiljan Hendriks, Joost te Riet, Katarina Wolf

**Affiliations:** 1 Department of Cell Biology, Radboud University Medical Center, 6525 GA Nijmegen, The Netherlands; 2 Department of Tumor Immunology, Radboud University Medical Center, 6525 GA Nijmegen, The Netherlands; 3 Department of Mathematics, School of Mathematical and Physical Sciences, University of Sussex, Falmer, Brighton BN1 9QH, UK; 4 Meinig School of Biomedical Engineering, Weill Institute for Cell and Molecular Biology, Cornell University, Ithaca, NY 14853, USA

**Keywords:** tumour cell, migration in confinement, speed oscillation, nuclear shape change, cell cycle, chromatin condensation

## Abstract

Directional cell migration in dense three-dimensional (3D) environments critically depends upon shape adaptation and is impeded depending on the size and rigidity of the nucleus. Accordingly, the nucleus is primarily understood as a physical obstacle; however, its pro-migratory functions by stepwise deformation and reshaping remain unclear. Using atomic force spectroscopy, time-lapse fluorescence microscopy and shape change analysis tools, we determined the nuclear size, deformability, morphology and shape change of HT1080 fibrosarcoma cells expressing the Fucci cell cycle indicator or being pre-treated with chromatin-decondensating agent TSA. We show oscillating peak accelerations during migration through 3D collagen matrices and microdevices that occur during shape reversion of deformed nuclei (recoil), and increase with confinement. During G1 cell-cycle phase, nucleus stiffness was increased and yielded further increased speed fluctuations together with sustained cell migration rates in confinement when compared to interphase populations or to periods of intrinsic nuclear softening in the S/G2 cell-cycle phase. Likewise, nuclear softening by pharmacological chromatin decondensation or after lamin A/C depletion reduced peak oscillations in confinement. In conclusion, deformation and recoil of the stiff nucleus contributes to saltatory locomotion in dense tissues.

This article is part of a discussion meeting issue ‘Forces in cancer: interdisciplinary approaches in tumour mechanobiology’.

## 1. Introduction

Cell migration is an essential process during development, tissue maintenance and immune function, but is also of importance during pathological cell invasion, including cancer metastasis [1]. Whenever cells (of neoplastic origin) move through connective tissue which consists of spatially and mechanically complex three-dimensional (3D) protein meshworks [2], they respond to mechanical confinement by changing shape. *In vivo*, migrating cells encounter physical challenges composed of microstructural extracellular matrix (ECM) networks and tissue tracks with 1–30 µm width, which are comparable to or smaller than the cell diameter of migrating cells ranging from 8 to 15 µm [3,4]. The nucleus is the largest and stiffest cell organelle that reaches around 2- to 10-fold higher stiffness values than the surrounding cytoplasm [5,6] and plays a pivotal role in cell deformation. Accordingly, under ECM space conditions matching the nuclear diameter, or by cell-derived proteolytic tissue degradation to create a low resistance path that matches the thickest cell diameter, cell migration is not impeded [4]. By contrast, in confining ECM environments, the nucleus will deform and adapt to the constraints of the ECM, but at the same time also form a mechanical obstacle which will gradually slow down migration [4,7–9].

The nucleus is both positioned and transported by the coordinated functions of the leading edge protrusion, integrin attachment to the surrounding substrate, contractile actin cytoskeleton fibres, microtubules and the linker of cytoskeleton and nucleoskeleton (LINC) complex physically linking the chromatin to the cytoskeleton. This leads to alternating force build-up at the front, and contraction of the rear concomitant with rear end integrin de-attachment from ECM, which will move the nucleus forward. Thus, alternating cycles of mechanical pulling and pushing act upon the nucleus during cell migration [4,7,10–12]. Previously, we have proposed a multi-step translocation cycle of the nucleus during confined three-dimensional (3D) cell migration consisting of (I) pressure application by the external constraint onto the nuclear membrane in the direction of migration, (II) beginning deformation of the nucleus by the formation of a local prolapse slowing down migration, (III) gliding of the compressed and deformed nucleus through the pore and (IV) rear release connected to rapid forward pushing and rounding (recoil) of the nucleus [13]. Implicit to this cyclic process, the migration delay during phase II is consistent with the ‘physical barrier’ function of the nucleus and might represent a phase of storage of deformation energy, which is released as propulsive energy during phase IV, leading to short phases of increased migration. It is, however, not clear whether and to what extent and by which physical characteristics the nucleus contributes to the acceleration of cell migration.

The overall stiffness, or elasticity, of the nucleus in an intact cell is dependent on a number of structural determinants, including A-type lamins that are part of the nuclear lamin network underlying the double nuclear membranes, as well as the organization of chromatin [14–20]. The chromatin packing state is variable and changes according to transcriptional needs, the extent of DNA repair, and the cell cycle phase, particularly when the diploidic G1 stage undergoes DNA replication in S phase to reach the tetraploidic G2-phase stage. Each of these events involves changes in DNA organization, with transient and reversible conversions of dense heterochromatin to more open euchromatin by histone acetylation or de-methylation [21–23]. These two latter processes lead to chromatin decondensation, nuclear softening and concomitant size increase [24–27]. Conversely, chromatin condensation increases nuclear compaction and stiffness in conjunction with a smaller size, as well as migration in wound healing and transwell chamber assays [20]. However, how these complex physical alterations of the nuclear interior impact shape change and migration in 3D confining environments remains to be assessed. In addition, it is unclear how softening of the nucleus, i.e. by lamin A/C reduction [28–30], affects nuclear recoil when cells exit a confined space.

Here, we used independent strategies of altered nuclear elasticity by chromatin condensation state and lamin A/C expression, and probed how these affect nuclear deformation and reshaping, as well as related migration patterns and rates. By comparing chromatin-compacted G1 cell-cycle phase cells to cells migrating during interphase or S/G2 phase, or by treating cells with the chromatin decondensating compound Trichostatin A (TSA), we co-registered speed oscillations and nuclear deformations. In addition, we developed and adapted a computational approach to calculate fluctuations of nuclear shapes during each migration phase. Our results indicate that deformation of the elastic nucleus during the passage of a constriction generates a recoil event that transforms into nuclear reshaping by rounding and, simultaneously, boosts nuclear propulsion and instantaneous migration velocity.

## 2. Results

### (a) Stiff G1 cell-cycle phase cells maintain fast migration in confinement by shape change

**Figure 1.**
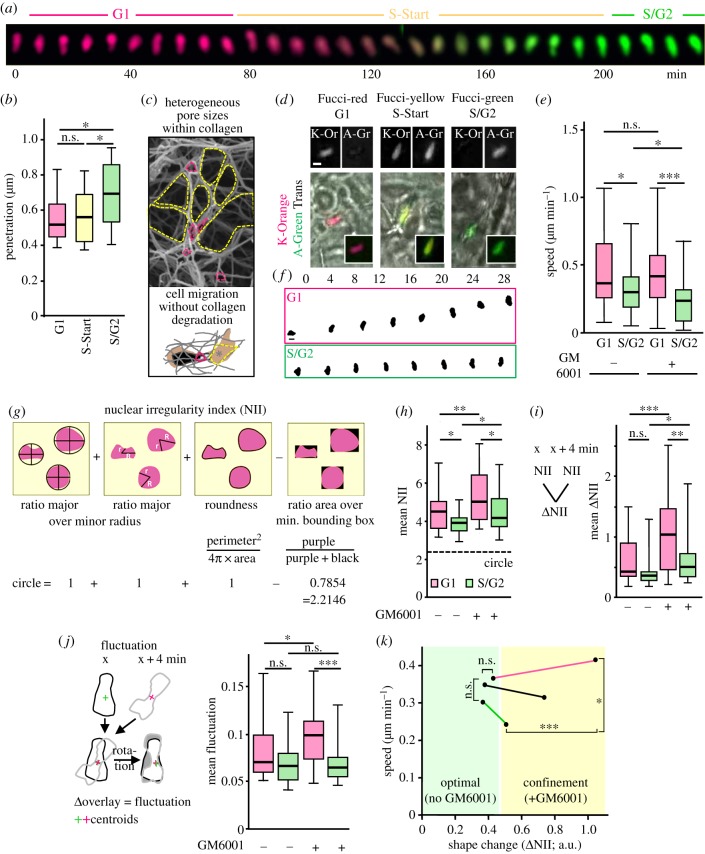
Maintenance of migration through confining pores associates with shape change of the small and rigid G1 cell-cycle phase nucleus. (*a*) Sequence of a cell nucleus from a Fucci-positive HT1080 cell progressing through the cell cycle, as indicated by changing colour coding over 4 h. This sequence is part of electronic supplementary material, figure S2 and Movie S1. (*b*) Quantification of cell deformation (penetration) after 2 nN contact force by a bead-coupled cantilever connected to an atomic force microscope (quantified from the approach curve; electronic supplementary material, figure S1D). *N* = 3; 25–32 cells per indicated cell-cycle phase. (*c*) Top, example of collagen matrix (monitored by scanning electron microscopy as shown in Wolf *et al.* [4]) showing small pore areas (confining; marked in pink), adjacent to large pores (marked in yellow). Bottom, cartoon depicting cell and nucleus in collagen of heterogeneous, colour-coded pore areas, including open space for translocation of cell and nucleus (asterisk). (*d–f*) HT1080-Fucci cells migrated in collagen (1.7 mg ml^−1^) in the presence of matrix metalloproteinase (MMP) inhibitor GM6001 (except where the absence of GM6001 is indicated), as monitored by pathway microscopy. (*d*) Examples of migrating cells at different indicated cell-cycle phases. Image bar, 10 μM. (*e*) Averaged migration speed of single cells from Fucci-red or -green populations from movies of 5–24 h length at indicated conditions. Because the S-start phase covers a short, around 2 h, time period, from here on and in all further experiments G1-phase cells were compared with S/G2 cells only. *N* = 2–3; 40–65 cells per condition. (*f*) Nuclear segmentation of migrating HT1080-Fucci cells. Time in minutes as indicated. (*g–j*) Nuclear shape analysis. (*g*) Top, schematic illustration of the components used for the calculation of the nuclear irregularity index (NII), adapted from Filippi-Chiela [32]. Bottom, calculation of all components that describe a circle, resulting in a NII of 2.2146. (*h*) Mean NII values per cell were computed and calculated from each nuclear shape sequence over time. (*i*) Left, scheme for calculation of the difference between subsequent NIIs as ΔNII, where rapid shape change of the nucleus results in a high ΔNII value. Right, mean values of ΔNII from each nuclear shape sequence over time. (*j*) Nuclear fluctuation analysis, sketching the analysis procedure (left), and mean values of the fluctuations from each nuclear sequence to the next (right). In (*h–j*), mean value per cell over 36–316 min; *n* = 3; 26–38 cells per condition. (*k*) Summary of speed as a function of nuclear shape change (ΔNII) in optimal and confining collagen conditions (data are from (*e,i*). Dots connected by red line represent G1 cycle cells; by green line, S/G2 cycle cells; by black line, G1 and S/G2 cycle cells together. (*b,e,h,i,j*) Black horizontal lines, boxes and whiskers show the medians, 25th/75th, and 5th/95th percentile (*b*, 10th/90th percentile), and ***, *p* ≤ 0.001; **, *p* ≤ 0.01; *, *p* ≤ 0.05; ns, non-significant (Mann–Whitney test).

To investigate the effect of the cell cycle on 3D migration, we generated HT1080 cells stably expressing Fucci, a fluorescent ubiquitination-based cell-cycle indicator [31] (electronic supplementary material, figure S1A). The expression of Cdt1-Kusabira-Orange2 in G1 cells and Geminin-Azami-Green1 in S/G2/M cells enabled us to visualize the forward progression of the cell cycle as G1, S-Start and S/G2 phase by detecting either only red, overlapping red and green, or only green signal (figure 1*a*). From all cells of a subconfluent 2D culture that expressed fluorescence, on average 28% of the cells were in G1, 14% in G1-to-S transition and 58% in S/G2 phase, detected microscopically and generally confirmed by flow cytometry (electronic supplementary material, figure S1B). In accordance with earlier findings [26,31,33], the nuclear area of cells during cycle progression from G1 to S/G2 phase increased by 23% in cell culture or 53% after embedding in 3D collagen matrices, respectively (electronic supplementary material, figure S1C).

To determine the rigidity of the nucleus during cell-cycle progression in HT1080 cells, we probed cultured Fucci sensor-expressing cells by atomic force spectroscopy (AFS), using a bead-coupled cantilever (electronic supplementary material, figure S1D) [34]. With transition from G1 to S/G2 phase, cell deformation increased by 1.4-fold (figure 1*b*) and, consequently, the calculated elastic moduli in G1 cells (991 ± 415 Pa) decreased by 23% after transit to S/G2 phase (767 ± 455 Pa), in line with previous data using HeLa cells [26]. According to increased chromatin condensation in G1 when compared with S/G2 cells, we calculated a 1.4-fold decrease in dissipation energy in G1-phase cells (electronic supplementary material, figure S1D-G). Collectively, our findings confirmed that cells in G1 have stiffer and less viscous nuclei compared to cells in S/G2, consistent with a higher chromatin compaction status in the G1-phase nucleus.

Next, the impact of cell-cycle progression on HT1080 cell migration efficacy through collagen lattices was tested. During polymerization of collagen into fibrillar networks, interfibrillar spaces of heterogeneous size are formed. Pores that are smaller than the cross-section of the polarized cell body will confine the migrating cell, whereas adjacent, more open spaces will provide sufficient space for forward movement (figure 1*c*). Consequently, cell and nucleus will repeatedly deform during migration. In addition, many activated or cancer cell types, including HT1080 fibrosarcoma cells, express a range of matrix metalloproteinases (MMPs) such as MMP-14. These cells migrate through confining collagen matrices by proteolytic remodelling of collagen fibrils, creating paths with 50–100 µm^2^ pore areas that match the respective cell cross-sections [35]. In this setting, MMP broad-spectrum inhibitor GM6001 inhibits collagen degradation and therefore increases the deformation of cell and nucleus during migration through confining collagen of around 5–25 μm^2^ pore size [36]. To test in context how cell deformation and migration speed are altered with transit from G1 to S/G2 phase, migrating HT1080-Fucci cells were optically separated into Fucci-red, -yellow and -green (figure 1*d*). Proteolytic migration speed of a G1 phase cell population in 3D collagen lattices was 0.4 µm min^−1^ and reduced by 18% in S/G2-phase cells, whereas at conditions of confinement, migration in S/G2 cells was increasingly compromised by 42%, reaching 0.23 µm min^−1^ (figure 1*e*). A similar 50% deceleration in speed, and concomitant area increase, was confirmed during the migration of a single cell during transition from G1 to S/G2 phase (electronic supplementary material, figures S2A-B; C, two most upper graphs; electronic supplementary material, Movie S1). The reduced migration rates of the S/G2 cell population, especially in confinement, may result from complex nuclear properties, including increased nuclear size and chromatin decondensation during S-phase and prior to mitosis, combined with reduced cell adhesion and actomyosin-mediated stiffness [26]. We asked, however, how cells in G1 phase could maintain speed in confinement despite a comparably stiffer nucleus.

To gain insight into the extent of nuclear deformation relative to speed changes during migration in confinement, we thresholded nuclear shapes (figure 1*f*; electronic supplementary material, Movies S1, right section; S2) and quantified respective shape changes over time as nuclear irregularity index (NII; figure 1*g*) [32]. The NII determines the level of shape deviation of the nucleus from a circle, and was chosen over more widely used nucleus roundness measurements because of its higher sensitivity (electronic supplementary material, figure S3). Whereas nuclei in moving S/G2-phase cells were more roundish, nuclei in G1 phase adopted somewhat more irregular shapes, which further increased by the presence of GM6001 (figure 1*h*). The changes between these shapes over consecutive time points were similar during proteolytic migration but, when MMPs were inhibited, increased by 2.4-fold in G1 phase but only 1.3-fold in S/G2 phase cells (figure 1*i*). To support these findings on dynamic nuclear shape changes, we included nuclear fluctuation analysis (figure 1*j*, left; electronic supplementary material, Movie S3). This tool calculates the difference between consecutive nuclear shapes, and thus the shape change. To exclude errors owing to a directional change of a cell during migration, nuclear shapes were rotated for maximal overlap. Again, nuclei in G1 phase showed more profound shape fluctuations in the presence of GM6001 whereas S/G2 cell nuclei did not change their shape at all (figure 1*j*, right). In combination, these two methodologies complement each other for robust measurement of nuclear shape change, and are applicable to all types of shape changes of biological objects over time and location. Together, in contrast to S/G2 phase or mixed G1/S/G2 cell populations, G1-phase cells maintained migration speed in confinement, associated with elastic deformation of the nucleus (figure 1*k*).

### (b) Deformation–rounding cycles of the nucleus correlate with speed oscillations

**Figure 2.**
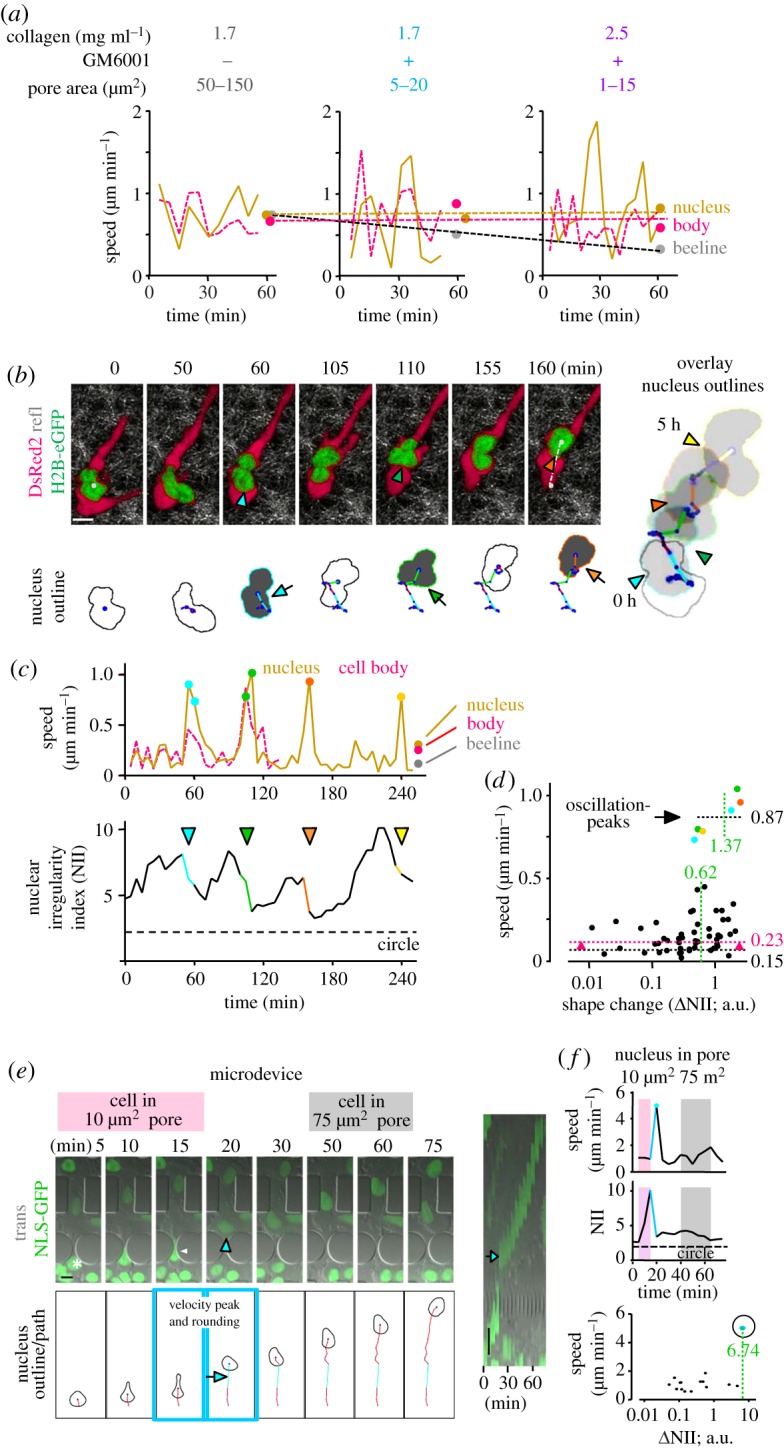
Speed oscillation and rapid nuclear rounding during cell migration in confining pores. HT1080 fibrosarcoma cells moved either in collagen (*a–d*) or in a synthetic microdevice (*e,f*). (*a*) Cells migrated in collagen of increasing density and the absence or presence of GM6001 as indicated, resulting in depicted decreasing effective pore areas (top; [36]). From each cell, speed of nucleus and cell body was quantified from the migration tracks from the centre of the nucleus as well as the cell body that often demonstrated synchronous speed peaks. Overall migration speed was measured as ‘beeline’ between the centre of the nucleus from the first and last image as depicted in electronic supplementary material, figure S4 and normalized over 55 min. (*b–d*) Oscillatory speed peaks of both cell and nucleus coincide with nuclear rounding. (*b*) Upper row, left, sequences of HT1080 dual-colour cell moving within high-density bovine collagen (3.3 mg ml^−1^) monitored by confocal microscopy at 37°C at 5 min intervals [13]. White dots at first and last image and dotted white line indicate position and beeline of the migrated nucleus. Lower row, left, outlines of the nucleus generated from H2B-eGFP signal, with centroids (blue dots) and centroid-connecting movement trajectories in red. Long trajectories indicated in cyan, green and orange and by arrows represent peak movements, and respective nuclear roundings are marked by coloured outline and grey area. Right, overlay of the first and all rounded outlines, and trajectory of the nucleus over 5 h (corresponding to electronic supplementary material, Movie S4). (*c*) Upper graph, step-to-step and average speed quantification from the movement trajectories of moving cell body and nucleus from (*b*), as well as the beeline of the migrated nucleus. Repeated speed peaks (oscillations) of the nucleus are indicated by respective coloured dots. Lower graph, corresponding repeated phases of nuclear rounding measured as NII, indicated by respective colours and arrowheads. (*d*) Speed as a function of ΔNII per time step (=dot) of the moving nucleus shown in (*b*), and quantified in (*c*). All numbers and dotted lines in black and green indicate medians of speed and ΔNII, respectively, for either coloured oscillation peaks or all remaining dots. Dotted line and number in pink indicate speed median from all dots. (*e*) Migration in microdevice of 10 and 75 µm^2^ pore areas (corresponding to electronic supplementary material, Movie S5, part 2). Left, upper row, sequence of migrating cell over indicated time. White arrowhead, deformed nucleus in pore. Arrowhead in cyan indicates rounding. Lower row, nuclear outlines with centroids as blue dots and trajectories in red. Arrow indicates long trajectory in cyan that corresponds to nucleus rounding. Right, kymogram visualizing rapid forward movement (arrow) after transmigration of narrow pore. (*f*) Speed and corresponding shape, with colour coding indicating respective pores in (*e*). Bottom, speed–nuclear shape change relationship; number in green, ΔNII value for peak oscillation. Reprinted modified images in (*b*) are with permission from Elsevier [13]. All image bars, 10 µm.

To approach how deformation of the nucleus may support cell migration, the velocity of cell body, nucleus and the overall migration efficiency were recorded together with the nuclear morphology. To collagen lattices of decreasing pore sizes from around 100 to 10 µm^2^, HT1080 cells responded with a gradual decline of overall migration efficacy (‘beeline’; figure 2*a*), consistent with our previous report [4]. At the same time, the speed of both nucleus and cell body remained unperturbed owing to increasing oscillations (figure 2*a*), with the nuclear shape increasingly co-fluctuating (electronic supplementary material, figure S4, middle column). These speed and shape fluctuations corresponded to lateral ‘swinging’ of both nucleus and cell relative to the main axis of movement (figure 2*b*; electronic supplementary material, Movie S4; figure S4, left column). In parallel, increasing nuclear deformations preceded rapid nuclear rounding (figure 2*b*, grey filled nuclear outline; *c*, bottom). At oscillation peaks, nuclear shape change doubled from 0.62 to 1.37 (figure 2*d*), while the cell body remained elongated. Every nuclear rounding event was precisely accompanied by an up to sixfold accelerated speed peak, each moving the nucleus by about half of its diameter forward (figure 2*c*, top; *b*, right). Accelerations versus remaining steps were on average 0.87 versus 0.15 µm min^−1^, increasing overall migration of the nucleus by 50% (0.23 µm min^−1^; figure 2*d*), as well as similarly the cell body. To support these findings in a migration model that enabled the visualization of the nucleus through pores of defined shape and size, a polydimethylsiloxane (PDMS)-based microdevice was used [37]. As in 3D collagen, migration through 10 µm^2^ pores was followed by nuclear rounding and concomitant speed acceleration, whereas movement through 75 µm^2^ pores lacked both nuclear shape change and speed oscillation (figure 2*e,f*; electronic supplementary material, Movie S5). Acceleration was several times higher as in collagen and possibly, owing to the higher elastic energy stored in the 5 µm^2^ small nuclear deformation, the result of a higher propulsive energy release into the barrier-free spacing following the tight pore. Taken together, cell passage through extracellular porous confinement coincides with nuclear deformation and is followed by piston-like acceleration of the nucleus together with transient rounding.

### (c) Saltatory propulsion of the rounding cell nucleus during migration in confinement is increased in G1-phase cells

**Figure 3.**
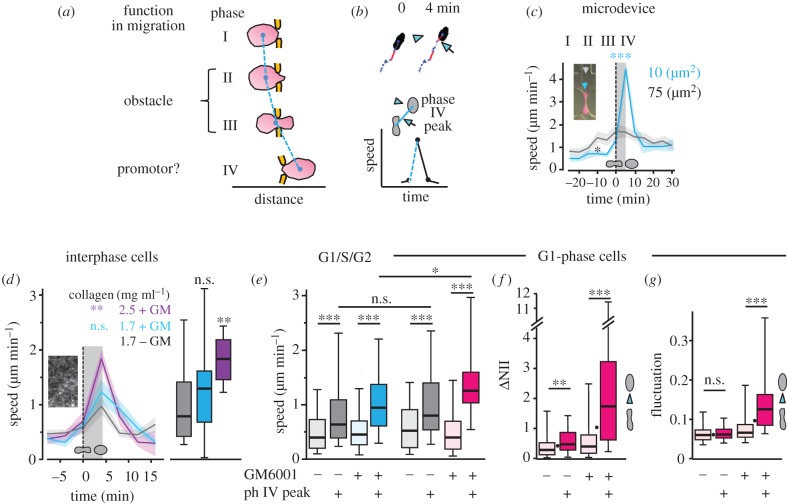
Characterization of ‘phase IV’ peak events. (*a*) Concept of nuclear deformation phases during cell migration through narrow pores. Adapted from Friedl *et al.* [13]. (*b*) Cartoon depicting selection scheme for phase IV speed peak values connected to nuclear rounding as shown in (*c*,*d*). (*c*) Speed of nuclei before (phase I and II), during (phase III) and after (phase IV) transmigration of pores of 75 µm^2^ (grey) and 10 µm^2^ (cyan) cross-section from microdevice. Vertical dotted line indicates time point when nuclei passed the centre of the pore (see inset), and grey shadowed area indicates phase IV. Solid lines represent mean, and shadowed coloured area ± s.e.m.; 16 and 18 cells per condition, *n* = 3. *p*-value was calculated for speed peaks at 5 min after pore passage of 10 µm^2^ when compared with data from 75 µm^2^ pores. Asterisk indicates decreased speed during passage of small when compared with large pores. (*d*) Quantification of phase IV speed peaks in collagen of decreasing effective pore size. The onset of each speed peak event was normalized to 0 min (dotted vertical line) and calculated based on speed increase in the moment of nuclear rounding (see black boxes in electronic supplementary material, figure S4,A-C, middle panels; S5A,B). *N* = 1–3; graphs are superimposed from 8 to 25 respective events from each 5 to 8 cells per condition monitored at high resolution; mean (coloured solid lines) ± s.e.m. (shadowed coloured areas). Grey shadowed area indicates phase IV event. Values of the peak speeds are also displayed as box plots. *p*-value was calculated for speed peaks at 4 min after pore passage, when compared with data from 1.7 mg ml^−1^ collagen in the absence of GM6001. (*e–g*) Depiction and analysis of either phase IV (strong colours) or all remaining non-phase IV events (light colours) from G1 cell-cycle phase cells (pink and grey), or mixed G1/S/G2 cell-cycle cells (blue and grey; *e*, left) after migration in collagen (1.7 mg ml^−1^) and GM6001 where indicated. Analysis of (*e*) speed, (*f*) ΔNII as shown in figure 1*i*, and (*g*) nuclear fluctuation, as shown in figure 1*j*, with the difference that instead of a mean value per cell over time, values from each time point of a moving cell were defined and separated into phase IV peak and remaining events selected as in (*b*). The single dots in (*f,g*) depict the medians from the G1-phase cell results from figure 1*i,j*. (*e*, left; G1/S/G2 cells) *N* = 1–3; 50–79 phase IV peak events and 363–387 remaining events were analysed from nuclear sequences of 16–21 cells per condition. (*e*, right – *g*, G1 cells) *N* = 1–3; 24–53 phase IV peak events and 231–291 remaining events were analysed from nuclear sequences of 8–10 cells per condition. In (*d*–*g*), data are depicted as horizontal lines, boxes and whiskers for medians, 25th/75th and 5th/95th percentile. For all experiments, ***, *p* ≤ 0.001; **, *p* < 0.01; ns, non-significant (both Mann–Whitney and Kolmogorov test).

Sustained migration in confined 3D environments in G1 cell-cycle phase (figure 1*k*) is in line with a proposed promoting effect of chromatin condensation on cell migration [20,38], yet the mechanism of speed gain remains unclear. The above-described data suggest that the nucleus acts as obstacle during cell passage through small pores during phase II, but in addition supports migration in phase IV by pushing through the confinement and regaining roundish shape (figure 3*a*). To further characterize this last step of the transmigration process, we mapped nuclear speed and corresponding nuclear shapes (NII) over time, normalized to the moment of nuclear rounding (phase IV peak) (figure 3*b*; electronic supplementary material, figure S5A,B; Movies S2, S5). This population analysis revealed, after a low-speed pore negotiation time, increased velocity peaks and, hence, speed oscillations for a population of single cells migrating through 10 µm^2^ pores in engineered microdevices, whereas no peaks were generated in 75 µm^2^ pores (figure 3*c*), consistent with data shown previously [37,39]. A similar stepwise peak acceleration, even though at lower velocity levels, was obtained with confinement in collagen (figure 3*d*). In G1 phase when compared with aggregated G1/S/G2 cells, speed peak events were again identified (electronic supplementary material, figure S5A,B). The individual step-to-step speeds that G1/S/G2 cells performed during phase IV in confinement (blue box plot in figure 3*e*) were two times higher than the remaining migration steps, but were exceeded by around 25% in G1-phase nuclei (red box plot in figure 3*e*). Similarly, respective nuclear shape changes (ΔNII, fluctuation) during phase IV peaks were two- to fivefold higher in confinement when compared with remaining events and to phase IV peaks in cells migrating in collagen in the presence of proteolysis (figure 3*f,g*). This principle also held true when relating shape change and speed in confinement for a single cell-cycle progressing cell (electronic supplementary material, figure S2C,D and Movie S1). Together, despite overall deceleration of cell migration in strongly confining environments, phase IV peaks support intermittent acceleration, and thereby sustained migration, in G1/S/G2 interphase cells. These accelerations are further increased in G1 cell-cycle cells only and, again, are associated with elastic deformation of G1-phase cell nuclei. By contrast, spatially optimized environments allow for effective speed throughout all phases of nuclear deformation, resulting in small or absent speed fluctuations.

### (d) Experimental chromatin decondensation reduces shape change and impairs migration

**Figure 4.**
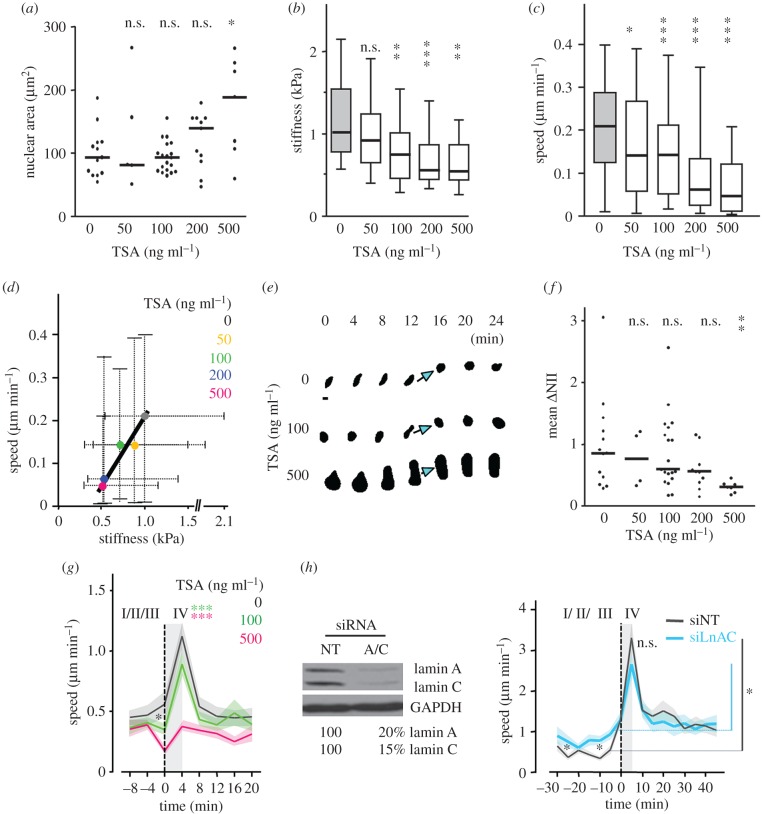
Altered stiffness, migration and reshaping of HT1080 cells and nuclei after TSA treatment or lamin A/C downregulation. Cells were pre-treated with indicated TSA concentrations or DMSO alone (indicated as 0 ng ml^−1^ TSA), and were either measured for elastic modulus (*b*) or migrated in collagen (1.7 mg ml^−1^) in the presence of GM6001 (*a,c–g*). For all TSA experiments, except in (*b*) and (*c*), Fucci cells were used and G1-phase cells only were selected for analysis. (*a*) Nuclear areas after pre-treatment with TSA in collagen. Horizontal black lines show the medians. *N* = 1; 5–19 cells per TSA concentration. (*b*) Calculated stiffness at 1.5 nN contact force by bead-coupled cantilever probing using atomic force microscopy. *N* = 1–3; 14–37 cells per condition. (*c*) Mean cell migration efficacy per cell over 24 h with increasing concentrations of TSA. Cells that underwent mitosis during the recording were excluded from the analysis. *N* = 3; 66–90 cells per condition. (*b,c*) Horizontal black lines, boxes and whiskers show the medians, 25th/75th, and 10th and 90th (*b*) and 5th/95th (*c*) percentiles. (*d*) Correlation of stiffness with migration efficacy, using the medians and whiskers from (*a,b*). *R*^2^ = 0.92. (*e*) Representative segmented nuclear sequences from migrating G1-phase (Fucci-red) cells at indicated time steps, in correspondence to Movie S6. Arrows indicate phase IV peak events based on speed increase and concomitant nucleus rounding. Bar, 10 µm. (*f*) Nuclear shape change during migration by TSA. Mean ΔNII per cell migrating over 0.3 to 10 h. Zero means no changes between subsequent nuclear shapes 5–19 cells per condition, shown as dots. Horizontal black lines show the medians. (*g*) Speed peaks, as in figure 3*d*, at indicated TSA concentrations. Graphs are superimposed from 22 to 38 respective events from each 7 to 19 cells per condition; *n* = 1. Mean (coloured solid lines) ± s.e.m. (shadowed coloured areas). Asterisk indicates decreased nuclear speed after TSA treatment before phase IV peak. (*h*) Left, lamin A/C expression intensity by western blot after transient downregulation by indicated siRNA (each 10 nM). Right, speed peaks in cells treated with non-targeting and lamin A/C siRNA after transmigration of 10 µm^2^ pore in a microdevice. Asterisks indicate increased nuclear pore negotion speed after silamin A/C treatment before phase IV peak event. Each 21 cells per condition. (*g,h* right) Dotted vertical lines, speed peak at nuclear rounding; grey-shadowed areas, phase IV events. ***, *p* ≤ 0.001; **, *p* ≤ 0.01; *, *p* ≤ 0.05; non-significant *n*.s. (*a,b,c,f*, Mann–Whitney test; *g,h* Students *t*-test).

To directly test whether chromatin condensation can promote phase IV peaks for sustained cell migration in confinement, we treated cells with chromatin decondensating TSA. Consistent with nuclear swelling after chromatin decondensation [20], and confirmed here by a relatively low cell number, nuclear size in G1-phase cells increased after TSA pre-treatment in a dose-dependent manner, but not yet at a concentration of 100 ng ml^−1^ (figure 4*a*). Accordingly, nuclear elasticity decreased in a dose-dependent manner (from 1 to 0.6 kPa; figure 4*b*) [34], confirming data from HeLa cells [26]. Besides softening of the nucleus, TSA pre-treatment decreased migration speed in confinement by 33–80% (figure 4*c*) and speed correlated positively with nuclear stiffness (figure 4*d*). Congruent to lowered elasticity after chromatin decondensation, nuclei deformed more slowly during migration and their ability to change shape reduced by up to 60% compared to an untreated control (figure 4*e,f*; electronic supplementary material, Movie S6). Accordingly, TSA treatment dampened phase IV accelerations (figure 4*g*), which for a TSA concentration of 100 ng ml^−1^ were not owing to an increase in nuclear size, but owing to a softened nucleus and a possibly less dynamic microtubule network, leading to overall decreased migration rates in confinement. Treatment with 500 ng ml^−1^ TSA entirely inhibited speed fluctuations, which might, in addition, be owing to the greatly increased nuclear size. Together, the data indicate that speed acceleration for effective migration in confinement requires chromatin condensation linked to high nuclear elasticity.

Besides chromatin condensation state, lamin A/C contributes to nuclear rigidity regulation. Downregulation of lamin A/C softens nuclei and supports migration rates through small pores [28,40,41]; however, its contribution to nuclear acceleration in phase IV remains unclear. Depletion of lamin A/C by RNAi reduced protein expression by 80% and nuclear stiffness by one third (our AFS analysis; data not shown), and was associated with a 40% decreased ‘phase IV’ speed acceleration when compared with untreated cells (figure 4*h*). These data are in agreement with an increased deformation of the softened cell nucleus of lamin A/C-deficient cells when compared with control cells during and after passage through small pores [37]. The somewhat higher migration rates during the remaining phases I–III (see asterisk) are indicative for the previously shown increased migration rate of cells after lamin A/C downregulation in confinement [29,40–42]. Together, the data indicate that the nuclear deformation energy during high confinement is released into nuclear rounding during phase IV peaking and is proportional to lamin A/C expression- and chromatin condensation-mediated nuclear elasticity.

## 3. Discussion

Here, we investigated 3D migration patterns of HT1080 fibrosarcoma cells in confining collagen lattices and microfabricated migration devices. The data reveal that cells and their nuclei do not migrate at a continuous speed, but undergo speed oscillations with velocities that increasingly deviate from beeline migration with confinement. Similar data of increasing nuclear oscillations in confinement presented by Yamada & colleagues [43] motivated a role of the cell nucleus in acting as a piston pressurizing the nucleo-anterior cell compartment and this way driving cell migration forward. Our findings complement these data well, stating that nuclear fluctuations were caused by increasing nuclear deformations with confinement. This led to migration delay during the pore negotiation process in phases I–III, and was followed by a short speed-up phase during the last transmigration phase IV. Similar to speed oscillations, nuclear shape changes become higher in amplitude with increasing confinement, and this could be attributed to the postulated increasing nuclear recoil phenomenon in phase IV after passage through a narrow pore [13]. Nuclear speed acceleration peaks were further responsive to nuclear elasticity regulation. Whereas with high stiffness and smaller size (i.e. during the G1 cell-cycle phase) speed oscillations were increased, conditions that soften the nucleus (e.g. in S/G2 cell-cycle phase, after experimental chromatin decondensation by TSA or by lamin A/C depletion) dampened periodic speed gain after pore passage.

Generally, processes connected with chromatin decondensation (e.g. mediated by cell cycle progression or by respective pharmacological inhibitors) reduce migration, in accordance with a number of publications. Yano and co-workers tested migration of Fucci-positive MKN45 adenocarcinoma cell spheroids in confining Gelfoam^®^ gels, and observed the least effective outward migration for S/G2/M phase cells [44]. Likewise, by applying a novel nano-printing technology to generate pores of defined areas on a smooth grated surface, S/G2 cell-cycle cells showed less pore engagement and penetration efficiency when compared with M/G1 and G1 cell-cycle phases [26]. Furthermore, chromatin decondensation agents TSA as well as MTA, a general protein methyltransferase inhibitor, step-wise reduced B16 melanoma migration in a transwell membrane assay of 8 and 5 µm diameter pores [20], in accordance to our results at comparable pore size ranges in collagen. Of note, stathmin-regulated microtubule dynamics by acetylation/deacetyation positively or negatively, respectively, regulates 3D cell migration [45]. Tubulin deacetylation by HDAC6 is inhibited by TSA [46], and thus delayed migration rates are likely not only owing to chromatin decondensation and softening, but also owing to stabilization of microtubules. In their transwell assay, Gerlitz and colleagues observed that the migration rate through 8 µm diameter pores was reduced by TSA to around 75%, but through confining 5 µm pore diameters to 60%, and by MTA to 70% (8 µm) and 30% (5 µm pores). These data support a dual effect of TSA, as well as by MTA, namely on the cell's general migratory machinery as well as on the impact of nuclear deformation by confinement, again affecting migration.

In summary, we investigated the phenomenon of pore transmigration by speed fluctuation and nuclear recoil in the context of tumour cell invasion. It will be interesting to test whether and how such oscillations during phases of active migration are interdependent on cell rear end detachment, and whether they occur also in other biological contexts, i.e. during development, immune responses, or tissue repair. Together, this knowledge will have an impact on the general understanding of cell migration mechanisms in heterogeneous environments.

## 4. Material and methods

### (a) Fucci plasmid construction and lentivirus preparation

Plasmids pFucci-G1-Kusabira-Orange2 (Fucci-red) and pFucci-S-G2-M-Amazi Green1 (Fucci-green) [31] were purchased from MBL International. The IRES2 sequence was obtained by PCR using pIRES2-eGFP as template for primers 5′- GCG GAA TTC GTG TGT AGT ACT GTG TGT **GGA TCCGCC CCT CTC CCT C** -3′ and 5′-GCG CTC GAG GTG TGT CCC GGG GTG TGT **CCA TGG TTG TGG CCA TAT TAT C** -3′ (sequences in bold represent the IRES2 template-specific segments; underlined parts indicate restriction sites relevant for subsequent cloning steps). Obtained amplicon was digested with EcoRI and XhoI and inserted into pENTR-NotI/XhoI [47], creating pENTR-IRES2. An XmaI linker oligonucleotide (5′-TTA AGA CCC GGG TC-3′) was heated and allowed to cool to room temperature to obtain a heteroduplex oligonucleotide with AflII-compatible ends, which was subsequently introduced in the AflII site in pFucci-G1-Orange2. Consequently, the sequence encoding human Cdt1 coupled to monomeric Kusabira-Orange2 could be excised using XmaI and NcoI and transferred into XmaI/NcoI digested pENTR-IRES2, resulting in pENTR-IRES2-Orange2. Finally, the part encoding human Geminin coupled to monomeric Azami-Green1 was excised from pFucci-S-G2-M-Green by using EcoRI and HpaI, and inserted into EcoRI/ScaI digested pENTR-IRES2-Orange2. The resulting plasmid pENTR-Fucci was used as donor in a Gateway^®^ LR recombination reaction with destination vector pLenti6.2/V5-DEST™ (Invitrogen), yielding pLenti-Fucci; a single bicistronic lentiviral vector that results in dual-colour fluorescent labelling of live cells that are either in G1 (here referred to as Fucci-red) or S/G2/M (here referred to as Fucci-green) cell-cycle phase. All constructs were sequence-verified.

### (b) Cell lines, lentivirus production, cell culture and stable transduction of Fucci construct

The following cells were used: human HT1080 wild-type fibrosarcoma cells (ACC315; DSMZ Braunschweig; [4]); HT1080 dual-colour cells expressing cytoplasmic DsRed2 and nuclear histone-2B (H2B)–coupled EGFP [48]; HT1080 cells stably transfected with NLS-GFP [36] or H2B-mCherry; and HT1080 cells stably transfected with Fucci sensor. Cells were cultured in Dulbecco's modified Eagle medium (DMEM) supplemented with 10% fetal calf serum (FCS) containing L-glutamine (2 mM), sodium pyruvate (1 mM) and 100 U ml^−1^ penicillin, 100 µg ml^−1^ streptomycin and incubated at 37°C in a humidified 5% CO_2_ atmosphere. Before cell experimentation, the detached and only loosely attached mitotic cells were washed away to only include interphase cells into all further assays.

For the generation of Fucci-positive HT1080 cells, recombinant lentiviral particles were produced, where in 10 cm dishes with 95% confluent HEK-293FT cell cultures were transfected overnight with JetPRIME reagent (Westburg) and a mixture of pLenti-Fucci plasmid and ViraPowerTM Packaging Mix (Invitrogen) according to the manufacturer's instructions. The following day, medium was refreshed and 48–72 h later virus-containing medium was harvested, passed through a 0.45 µm pore size filter and stored at −80°C until further use.

For stable transduction, HT1080 cells were seeded into six wells at 20–30% confluency, and on the next day medium was aspirated and 1 ml of viral-containing medium was drop-wise added to the cells and incubated overnight. On the following day, the medium was replaced with fresh medium, followed by selection with 5 µg ml^−1^ blasticidine for 4–5 days after the transduction. Blasticidine was constantly kept on the cells during expansion, but removed 1–2 days prior to experimentation. Cells were sorted twice by flow cytometry for red or green fluorescence, resulting in a stable Fucci-positive cell population not higher, however, than around 70%. Dynamic imaging of 2D cultures revealed an approximated 90% rate of an appropriate cell-cycle-related temporal order of Fucci colours (data not shown). Cell growth characteristics of HT1080-Fucci-positive cells related to values measured for HT1080 cells previously, with cell cycle times of approximately 5 h in G1 phase, approximately 9 h in S/G2 phase and approximately 1.0 h in mitosis [34,49]. In addition, transfected cells remained functional with equal migration rates (0.2-0.4 µm min^−1^) when compared with parental HT1080 cells [4]. These characteristics validate the applicability of the Fucci construct in HT1080 cells for investigating cell-cycle-related functions.

For transient lamin A/C knockdown, cells were cultured in antibiotics-free supplemented DMEM in six-well plates (each 250 000 cells) for 24 h. Cells were treated with a pool of small interfering (si) RNAs consisting of four single RNAs each and targeting expression of lamin A/C or non-targeting (NT) negative control (10 nM; on-target plus, SMARTpool; Dharmacon). The forward 21-nucleotide siRNA sequences for the NT control were 5-UGGUUUACAUGUCGACUAA-3, 5-UGGUUUACAUGUUGUGUGA-3, 5-UGGUUUACAUGUUUUCUGA-3, 5-UGGUUUACAUGUUUUCCUA-3; for si*LMNA* the forward sequences were 5-GAAGGAGGGUGACCUGAUA-3, 5-UCACAGCACGCACGCACUA-3, 5-UGAAAGCGCGCAAUACCAA-3, 5-CGUGUGCGCUCGCUGGAAA-3. siRNAs were transferred into cells with Dharmafect 4 transfection reagent according to the manufacturer's protocol and cultured with antibiotics-free DMEM for 48 h prior to characterization and functional studies. Lamin knockdown efficiency was determined by electrophoresis and western blot analysis from whole-cell lysates (62.5 mM Tris–HCl; 2% w/v SDS; 10% glycerol; 50 mM DTT; 0.01% w/v bromophenol blue), followed by chemiluminescence detection (ECL detection kit; GE Healthcare) and densitometric analysis (Fiji ImageJ).

### (c) Analysis of the cell-cycle stage by flow cytometry

Flow cytometry was performed to determine the relative DNA amount in respect to Fucci colour within the cell population. Cultured HT1080 cells stably expressing Fucci marker were detached, re-suspended, and fixed with 500 µl 75% ice-cold ethanol for 1 h. Ethanol was carefully washed off and cells were incubated in 300 µl staining solution (1× PBS; 0.2 mg ml^−1^ RNase A, 1 µM DRAQ5) at 37°C for 30 min. Cells were measured on a CyAn ADP flow cytometer (Beckman Coulter) using spectral ranges 530/40 nm for Azami-Green1, 613/20 nm for Kusabira-Orange2 and 665/20 nm for DNA marker DRAQ5.

### (d) Probing nuclear mechanics by atomic force spectroscopy

Two days before AFS experimentation, 40 000 cells were seeded into a Willco dish in 1 ml DMEM/10% FCS and incubated at 37°C in a humidified 5% CO_2_ atmosphere. Twelve hours prior to the measurements, the medium was exchanged for 1 ml DMEM/10% FCS containing 10 mM HEPES (Gibco). Where indicated, cells were pre-treated with specified concentrations of histone deacetylase inhibitor trichostatin A (TSA, Sigma) 24 h before experimentation. Nuclear deformation measurements were performed using a Catalyst BioScope atomic force microscope (Bruker, Santa Barbara, CA, USA) combined with a three-channel confocal microscope TCS SP5 II (Leica, Mannheim, Germany) for simultaneous brightfield and epifluorescence imaging through a Hamamatsu (ORCA-05G) camera and an air objective (20×, 0.70 NA). Flexible NP-S cantilevers modified with a 10 µm diameter bead were mounted, calibrated by the thermal noise method [50], and subsequently located over the cell for repeated probing (three to five times) at an approach and retraction rate of 10 µm s^−1^ each with a pre-defined force of 15 nN. The registered force–distance (F-D) curves were transferred into force-indentation (F-δ) curves and used to calculate the penetration, stiffness and dissipation of the nucleus [34]. The stiffness was calculated by using a custom algorithm written in IgorPro 6 (Wavemetrics) for fitting the F-δ curves with the Hertz model for spheres in contact with a flat surface [51]. The energy dissipated during compression of the cell/nucleus was derived by using a custom algorithm written in Matlab (MathWorks, Inc.) that determined the areas underneath the approach and retraction curves. Subsequently, the residual integral was calculated (*E*_dissipation_ = *E*_approach_−*E*_retraction_), which represents the energy needed to deform the nucleus; i.e. force (Newton) × distance (meter) = energy (Nm = J). The adhesion energy *E*_adhesion_, measured as the area of the adhesion part in the retraction curve, was excluded because it is a measure of the stickiness of the bead probing the cell. Finally, to derive the relative dissipation energy during a probing cycle, *E*_dissipation_ was divided by the energy needed to deform the nucleus (*E*_approach_). The dissipation energy is an indicator for the viscous component of the visco-elastic properties of the cell. The elastic part of the cell is represented by its penetrability.

### (e) Three-dimensional collagen assays, time-lapse microscopy and quantitative cell tracking

Three-dimensional collagen lattices were prepared from acidic collagen solution (rat tail, Corning; or bovine, Advanced Biomatrix) supplemented with Minimal Essential Eagle's Medium (MEM; Sigma), HEPES and NaOH (for rat tail collagen) or bicarbonate (for bovine collagen) reaching a pH ∼ 7.4, and mixed with the cell suspension to a final density of 200 000 cells ml^−1^ in collagen of indicated source and concentration. Usually, a concentration of 1.7 mg ml^−1^ rat tail collagen was used, unless indicated otherwise. Where indicated, cells were pre-treated with TSA for 24 h, and it was confirmed that cells remained viable, as judged by the absence of abnormal cell morphologies or blebbing, and as further quantified by a propidium iodide assay, as detailed in Krause *et al.* [34]. A 100 µl cell-collagen mix was added to each well of a 96-well glass-bottom plate, allowed to polymerize at 37°C in humidified 5% CO_2_ atmosphere for 20–30 min, and was overlaid with medium. Where indicated, 5 µM GM6001 (Calbiochem) were added to both collagen and supernatant, and samples were monitored by temperature- and CO_2_-controlled live microscopy over up to 24 h in 4 or 5 min frame intervals. Combined brightfield/fluorescence imaging was performed on either an Okobab microscope (10×, NA 0.25, Nikon DiaPhot 300, a Hamamatsu ORCA AG CCD camera, an excitation and emission filter set for FITC and TRITC, and the 2D time-lapse software Attovision), or on a spinning-disc confocal microscope (Pathway 855; BD Biosciences; 20x/0.40NA air objective; excitation filter sets of 488/10 and 548/20 nm, and emission filter sets of 520/35 nm and 600/15 nm). Life confocal microscopy for high-resolution imaging of HT1080 cells was performed in time-lapse *z*-stack mode using a temperature- and CO_2_-controlled stage (37°C, 5%), and images were reconstructed as maximum intensity projections from all fluorescence and two reflection scans. Migration efficacy was quantified with the cell tracking program Autozell (v.1.0; Center for Computing and Communication Technologies (TZI), University of Bremen, Germany) of XY paths. The average speed per cell was calculated from the length of the migration path divided by time. Only viable cells that showed dynamic changes in cytoplasmic extensions and retractions were tracked and included into the analysis.

### (f) Microfluidic migration devices

Polydimethylsiloxane (PDMS) microfluidic devices were fabricated and used as previously described [37,52], with a migration chamber height of 5 µm and a pore width of either 2 or 15 µm, resulting in 10 and 75 µm^2^ cross-sectional areas. In brief, the migration devices were coated with 50 µg ml^−1^ rat tail type-I collagen (BD Biosciences) in acetic acid (0.02 N) overnight at 4°C and rinsed with imaging medium to remove the coating solution. After loading the cells, devices were incubated for at least 3 h to allow cell attachment before imaging. Microfluidic migration devices were imaged at 37°C on an inverted Zeiss Observer Z1 microscope equipped with a CCD camera (Photometrics CoolSNAP KINO) using a 20×/NA 0.8 air objective. The image acquisition was automated through ZEN (Zeiss) software with imaging intervals of 4 and 5 min collecting images for DIC and GFP. Time-series images were stabilized using a custom-written Matlab (Mathworks) script using features of the PDMS device as fiducials to compensate for the inaccuracy of the linear encoded microscope stage. Individual cells were tracked during the process of pore transmigration by using custom-developed Matlab software [53].

### (g) Image analysis

For image analysis, in-focus nuclear areas and dynamic changes in nuclear shapes over time were analysed using the program Fiji ImageJ, or Matlab with the in-house built-in software ITNA (Image and Trajectory Nuclear Shape Analysis; Sussex University). Image stacks were separated into individual channels, thresholded (mostly by automated thresholding algorithm Otsu) and nuclear outlines per time point were automatically collected. From these outlines, nuclear area, nuclear irregularity index (NII), and nuclear fluctuation were calculated. NII analysis was conducted by computing shape properties using the Imaging Processing Toolbox provided by Matlab [54]. To calculate fluctuation, the two consecutive images were overlaid with their centroids and a 1°stepwise full-circle (360°) rotation was performed to find the maximum overlap between these two shapes (electronic supplementary material, Movie S3). The resulting fluctuation number was computed through

$$\mathrm{Fluctuation}=\frac{A_{1}+A_{2}-2(A_{1}\cap A_{2})}{A_{1}+A_{2}},$$

where *A*_1_, *A*_2_ are the areas of the two consecutive nuclear shapes and $A_{1}\cap A_{2}$ are the maximum intersections found through the rotation. The value range of this fluctuation number is (0,1). The value 0 indicates complete overlap of the nuclear shapes, while the value 1 implies no overlap at all. Phase IV events were defined as a combination of a NII value decrease (nuclear rounding) and a speed increase as a function of time, and selected after visual inspection, and were depicted as speed as a function of shape change (ΔNII).

### (h) Statistics

Statistical analysis was performed on independent samples with non-Gaussian distribution, using both the two-tailed non-paired Mann–Whitney test and the two-sample Kolmogorov–Smirnov test. Independency of G1- and S/G2-phase samples was confirmed by the rejection of the null hypothesis (1% significance level; i.e. *p*-value = 0.01) and acceptance of the alternative hypothesis (99% confidence on the existence of a statistical significance between G1 and S/G2 phases).

## Supplementary Material

Figure S1. Characterization of HT1080 cells after stable transduction with Fucci vector

## Supplementary Material

Figure S2. Analysis of cell cycle transition-related nuclear parameters by imaging of a single HT1080-Fucci cell.

## Supplementary Material

Figure S3. Depiction of the advantage of nuclear irregularity index versus nuclear rounding calculation.

## Supplementary Material

Figure S4. Speed peaks and rounding of the nucleus increase with confinement.

## Supplementary Material

Figure S5. Selection and depiction scheme of phase IV speed peak/ nuclear rounding events.

## Supplementary Material

Movie S1. Spontaneous migration of mesenchymal HT1080-Fucci cells over different interphase cell cycle phases.

## Supplementary Material

Movie S2. Phase IV peak events in migratory G1 phase HT1080 cells.

## Supplementary Material

Movie S3. Illustration of the nuclear shape rotation method for fluctuation analysis.

## Supplementary Material

Movie S4. HT1080 dual-color cell in dense collagen: oscillatory migration and repeated rapid rounding of the nucleus.

## Supplementary Material

Movie S5. Migration of HT1080 cells through synthetic microdevice.

## Supplementary Material

Movie S6. TSA reduces cell migration efficacy and nuclear shape change.

## Supplementary Material

Supplementary figures and movies legends

## Data accessibility

Original data available from the Dryad Digital Repository: https://doi.org/10.5061/dryad.c806hk2 [55].
