# Supplementary figures and images for: Cell migration through three-dimensional confining pores: speed accelerations by deformation and recoil of the nucleus
Source: Philos Trans R Soc Lond B Biol Sci. 2019 Jul 1;374(1779):20180225. doi: 10.1098/rstb.2018.0225 (PMC6627020; doi:10.1098/rstb.2018.0225)

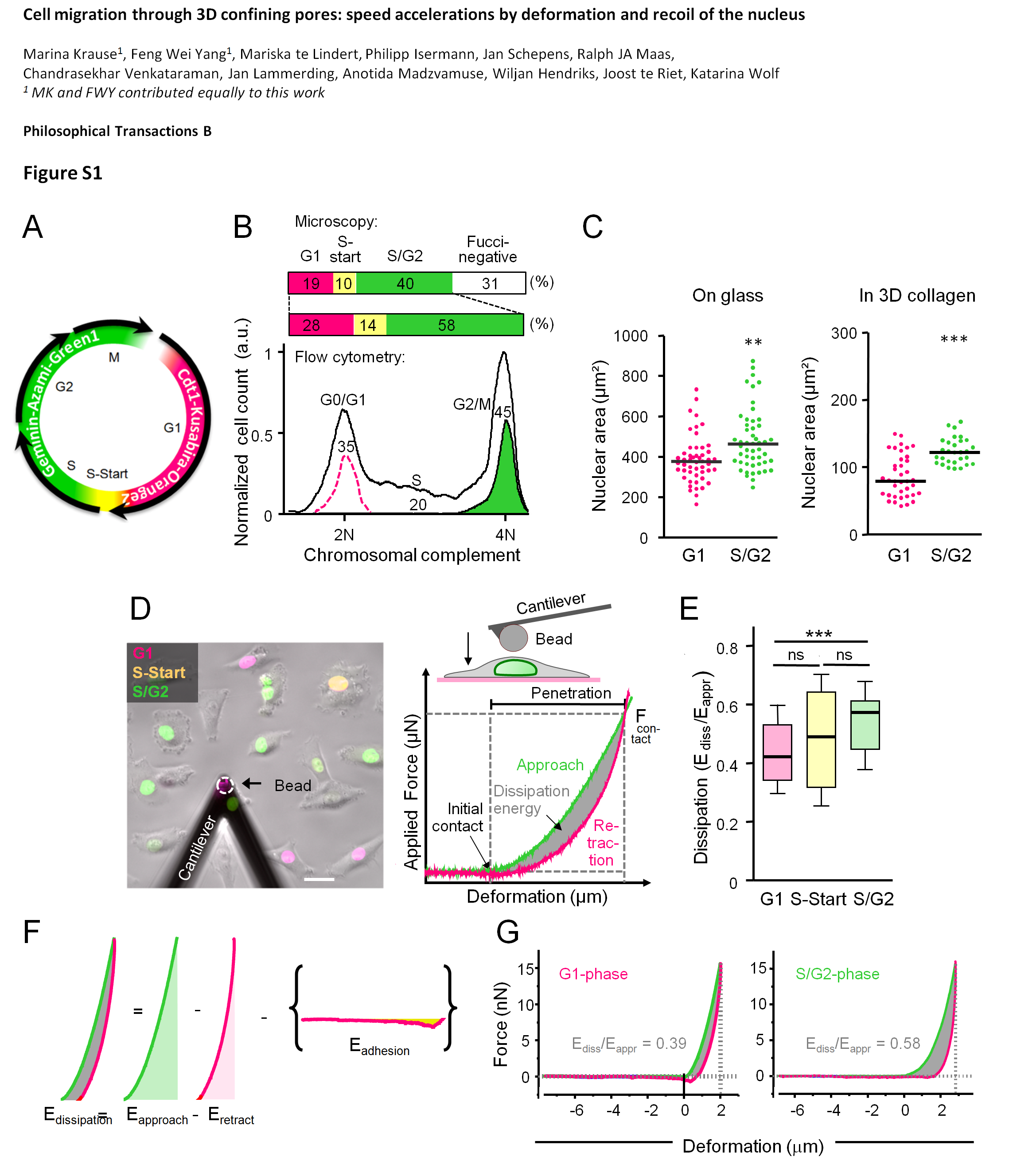

Supplement: Figure S1. Characterization of HT1080 cells after stable transduction with Fucci vector [file rstb20180225supp1.tif]

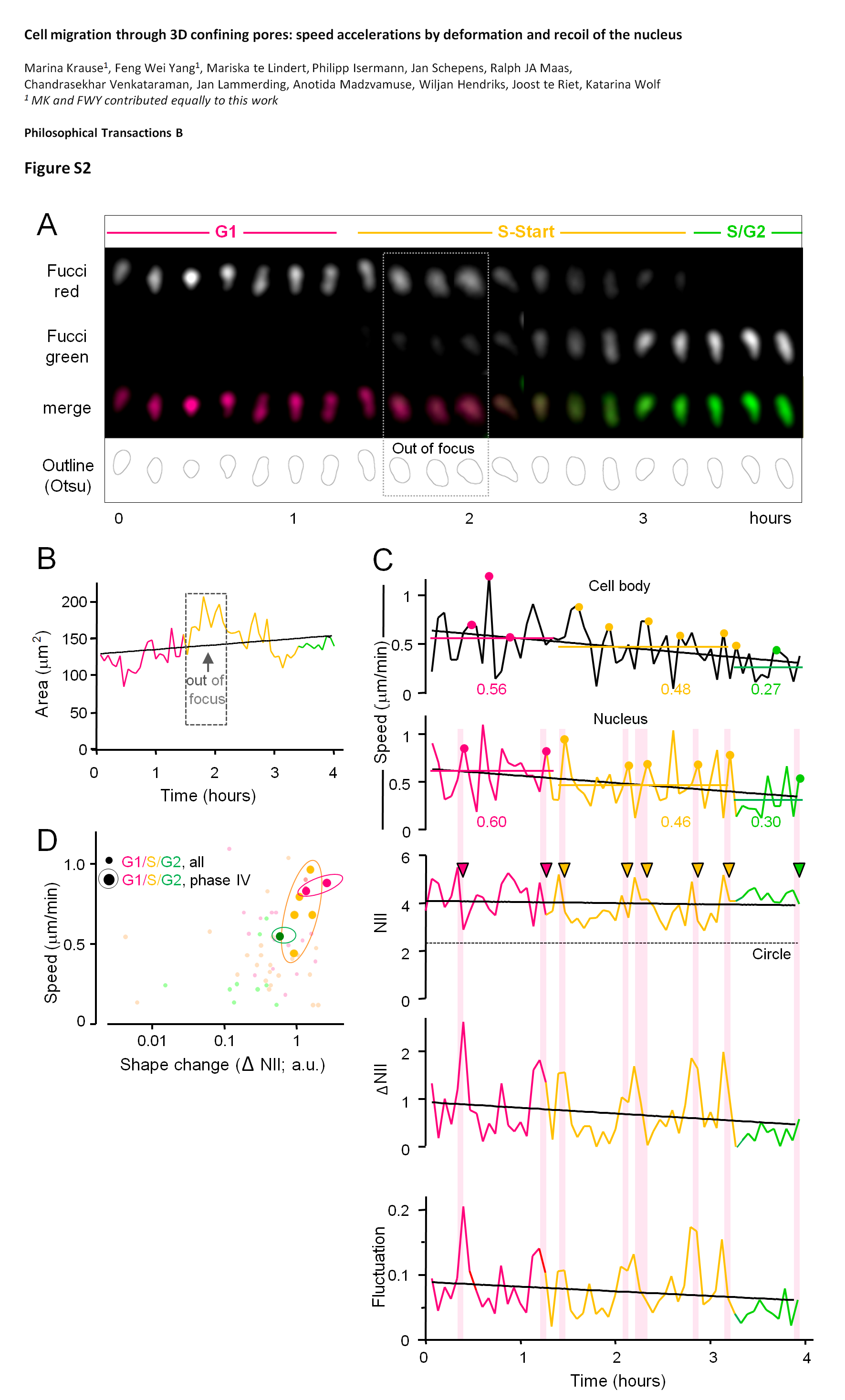

Supplement: Figure S2. Analysis of cell cycle transition-related nuclear parameters by imaging of a single HT1080-Fucci cell. [file rstb20180225supp2.tif]

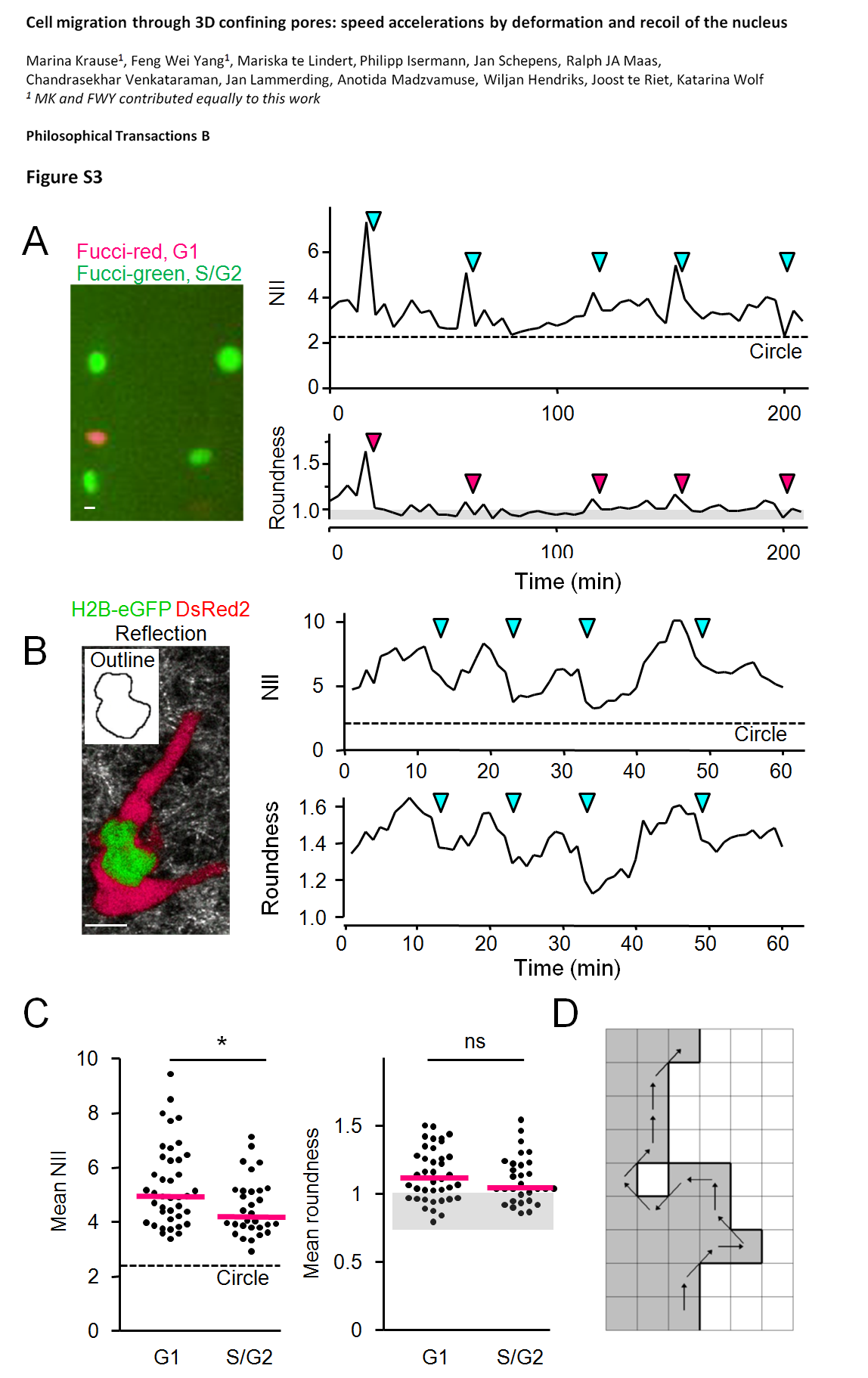

Supplement: Figure S3. Depiction of the advantage of nuclear irregularity index versus nuclear rounding calculation. [file rstb20180225supp3.tif]

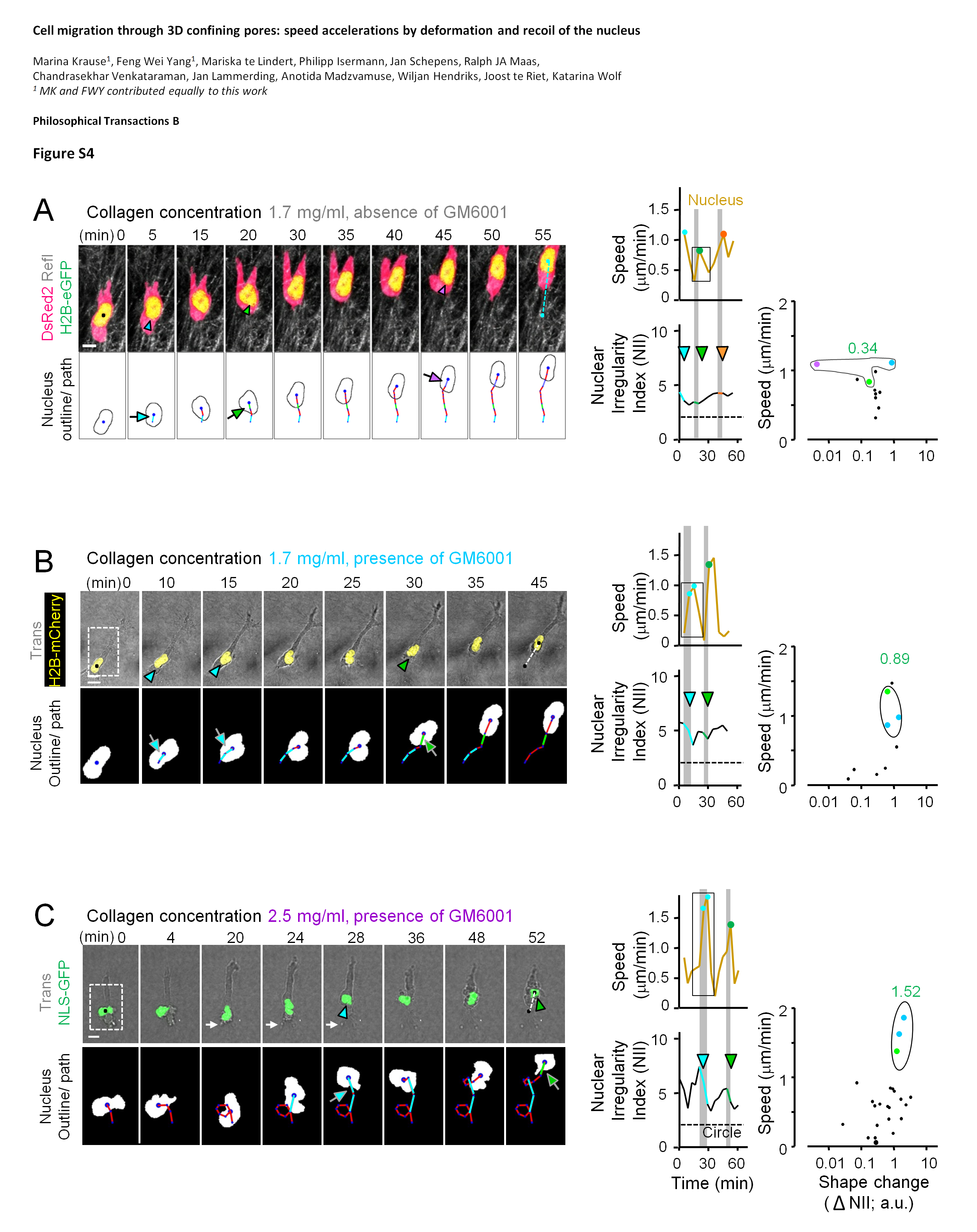

Supplement: Figure S4. Speed peaks and rounding of the nucleus increase with confinement. [file rstb20180225supp4.tif]

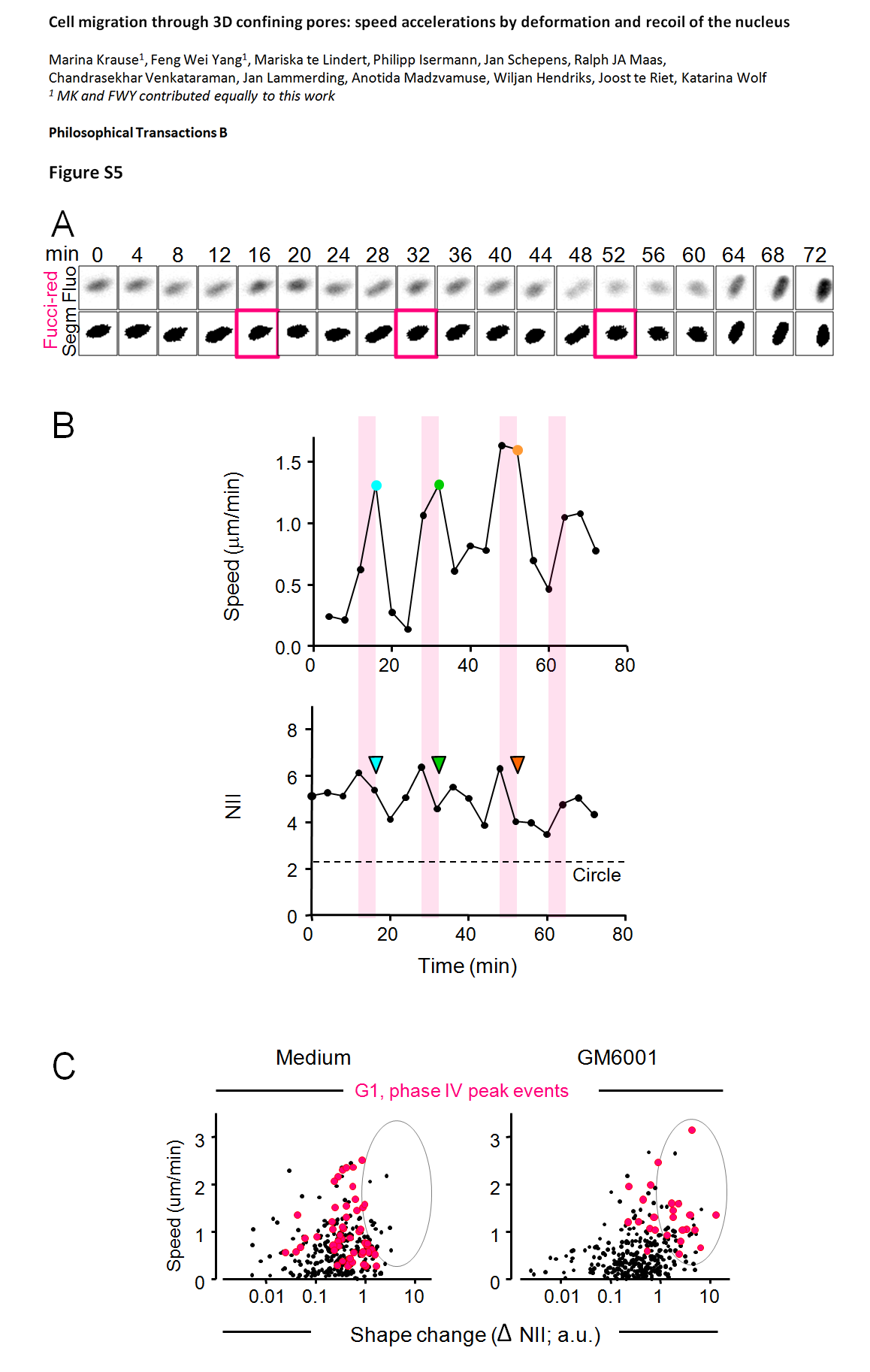

Supplement: Figure S5. Selection and depiction scheme of phase IV speed peak/ nuclear rounding events. [file rstb20180225supp5.tif]
