# Supplementary material for: Cell migration through three-dimensional confining pores: speed accelerations by deformation and recoil of the nucleus
Source: Philos Trans R Soc Lond B Biol Sci. 2019 Jul 1;374(1779):20180225. doi: 10.1098/rstb.2018.0225 (PMC6627020; doi:10.1098/rstb.2018.0225)
Supplement: Supplementary figures and movies legends [file rstb20180225supp12.docx]

**Cell migration through 3D confining pores: speed accelerations by deformation and recoil of the nucleus**

Marina Krause^a1^, Feng Wei Yang^b1^, Mariska te Lindert^a^, Philipp Isermann^c^, Jan Schepens^a†^, Ralph JA Maas^a^, Chandrasekhar Venkataraman^b^, Jan Lammerding^c^, Anotida Madzvamuse^b^, Wiljan Hendriks^a^, Joost te Riet^d^, Katarina Wolf^a^

^1^ MK and FWY contributed equally to this work.

^a^ Department of Cell Biology, Radboud University Medical Center, 6525 GA Nijmegen, the Netherlands

^b^ Department of Mathematics, School of Mathematical and Physical Sciences, University of Sussex, BN1 9QH, Falmer, Brighton, United Kingdom

^c^ Meinig School of Biomedical Engineering, Weill Institute for Cell and Molecular Biology, Cornell University, Ithaca, NY 14853, USA

^d^ Department of Tumor Immunology, Radboud University Medical Center, 6525 GA Nijmegen, the Netherlands

^†^ Deceased

**Supplementary figure legends**

**Figure S1. Characterization of HT1080 cells after stable transduction with Fucci vector.**

**(A)** Cartoon of fluorescence ubiquitination-based cell cycle sensor Fucci, adapted from the ThermoFisher Scientific website, demonstrating how cell cycle stages can be optically separated by color coding. The Fucci biosensor exploits the cell cycle-regulated proteolysis of Cdt1 and Geminin, two proteins involved in licensing of replication origins [1]. Ctd1- Kusabira-Orange2 expression is assigned to G1 phase (Fucci-red), which is degraded by the ubiquitin proteasome system when S phase is commencing. Reciprocally, Geminin-Azami-Green1 expression is assigned to the S, G2, and M phases (Fucci-green) and is rapidly broken down upon exit of mitosis. Consequently, cells express both colors (Fucci-yellow) only during the early S phase. Because before cell experimentation the detached and only loosely attached mitotic cells are washed away, green colored cells are in the following termed S/G2. (**B)** Distribution of cell cycle stages in HT1080 cells. Top, percentage of G1, S, and S/G2 phase distributions from 387 adherent cells from 2D cell culture. Bottom, flow cytometric analysis of an ethanol-fixed cell population. Chromosomal complementation is displayed as diploidic (2N) that refers to cells in G1 phase whereas the tetraploidic (4N) cell population refers to cells in S/G2 phase. Cells in S-start phase are distributed in between 2N and 4N, as chromatin duplication is not yet completed. Numbers within compartments are estimated fractions in percent. Back-gating confirmed that S/G2 Fucci-green cells (small peak in green) fill part of the 4N-related peak meaning that they have completed DNA duplication. Dotted pink line indicates the anticipated Fucci-red cell population, as Cdt1-Kusabira-Orange2 fluorescence was lost during fixation. (**C)** Nuclear areas in G1 and S/G2 phase, measured from cells either plated on glass or in collagen. Median, horizontal line. N=2, 51 cells per cell cycle phase (on glass); n=3; 38 and 29 cells per cell cycle phase (in collagen). **(D)** Principle of AFS-force measurements (see [2]). Image and cartoon show that prior to a measurement, a 10 µm-diameter-sized bead immobilized on an cantilever was positioned above the center of the nucleus. Cells were selected by epifluorescence microscopy mode for suitability by morphology and cell cycle phase. After cantilever-mediated approach, compression, and retraction from the cell, the resulting force-distance curves were plotted (graph). Image bar, 20 µm. **(E)** Relative dissipation energy, derived as the area between approach (E_appr_) and retraction (E_diss_), and divided by the energy needed to deform the nucleus (E_appr_). A value of 0 represents a purely elastic nucleus where approach and retraction curves overlap (the nucleus recoils completely, and all of the compression energy is recovered); increasing values indicate increasingly viscous material behavior, including plastic deformation and viscous dissipation of the energy required to compress the nucleus (the nucleus remains partially deformed after the cantilever has been retracted). N = 3; 30-32 cells per condition. Black horizontal lines, boxes and whiskers show the medians, 25^th^/75^th^, and 10^th^/90^th^ percentile.  **(F)** Cartoon depicting how the disspation energy was extracted from force-distance curves after a compression-relaxation cycle of a cell when probed by a bead-functionalized cantilever. **(G)** Two example curves of each nucleus in G1-phase and in S/G2-phase show the differences in dissipation indicative for the significant difference found in **(E)**. ***, P < 0.001; **, P < 0.01; ns, non-significant (Mann-Whitney test).

**Figure S2. Analysis of cell cycle transition-related nuclear parameters by imaging of a single HT1080-Fucci cell. (A)** Image sequence from a single migrating cell in collagen in the presence of GM6001, demonstrating a single cell cycle transition over 4 hours (see figure 1*a* and Movie S1). Upper lanes, single red, single green and merged channels. Lowest lane, nuclear outline produced in Fiji ImageJ by Otsu-thresholding. (**B,C)** Quantification of indicated parameters showing each of the gradual change over 4 hours. The up- or downwards trend is indicated by black lines obtained by the trendline function in Fiji, and this trend as well as all quantifications coincide with population data in figures 1 and S1. Pink, yellow and green colors indicate the different cell cycle phases as marked in A. **(B)** Quantification of increasing nuclear area during G1 to S/G2 phase transition. Grey shadowed area, out of focus region as marked in (A). (**C)** Top graph, speed fluctuation of cell body. All other graphs, from second top to bottom, quantification of speed and morphometric parameters (NII, delta NII, and fluctuation) of the nucleus, all decreasing from G1 to S/G2 phase. Speed- nuclear shape change relationship for identification of phase IV events are marked by vertical pink lines, and are shown as colored path segments in Movie S1. **(D)** Depiction of phase IV events (encircled) versus all events, color-coded per cell cycle phase. Quantification coincides with population data in figures 2*d* and S5C.

**Figure S3. Depiction of the advantage of nuclear irregularity index versus nuclear rounding calculation. (A)** Left, depiction of nuclei from 5 cells carrying the Fucci construct. Right, graphs show calculation of nuclear shape change for a nucleus from a migrating cell over time, using NII (formula introduced in figure 1*g*) and roundness. Colored arrowheads indicate nucleus rounding. Top, value for a circle is indicated; bottom, area in light grey indicating around 50% roundness values that oscillate around 1 [see explanation in **(D)**]. Thus, when a relatively low-resolution image is used, a single shape property like roundness can be unreliable, but NII with a combination of four shape properties is still robust, indicating the expected shape changes more clearly. **(B)** Left, depiction of nucleus from high-resolution imaging (figure 2*b*); right graphs, depicting NII and roundness and indicating convincing agreement. **(C)** Mean nuclear shapes calculated from single G1 and S/G2 phase cells monitored at low resolution. Difference is significant after calculation of NII, but not after calculation of roundness. Area in light grey indicates roundness values oscillating around 1. Because of roundness’ unreliability in low resolution imaging, NII was chosen over roundness. **(D)** Schematic illustration of how the nucleus perimeter was calculated within the calculation of roundness. Rather than a simple edge count method, a polygonal approximation was implemented. This approximation took into account the inward and outward corners, and straight lines. Different weights were assigned, since a digital representation of a shape can only be pixelated without smooth curves. Such an approximation with enough pixels improves the calculation of the perimeter, whereas less pixels entail an under-estimation of the perimeter with a value below 1. All image bars, 10 μm.

**Figure S4. Speed peaks and rounding of the nucleus increase with confinement.** HT1080 cells migrated in collagen of increasing indicated concentrations and the absence or presence of GM6001 (sequences and values correspond to figure 2*a*). **(A-C)** **Left column,** upper rows, sequences of migrating cells over indicated time periods and in indicated colors for cell body (DsRed2 or transmission signal), nucleus (H2B-eGFP, H2B-mCherry or NLS-GFP) and collagen reflection signal. Color-coded arrowheads indicate rounding even if marginal. Lower rows, nuclear outlines or segmentations, respectively, with centroids as blue dots and trajec-tories in red. Arrows indicate long, colored trajectories that correspond to nucleus rounding indicating phase IV peak events. **(B,C)** Dotted rectangle, inset where nucleus in bottom row was enlarged from. **(C)** White arrows, detachment of cell rear corresponding to velocity peak and rounding of nucleus. All bars, 10 μm. **Middle column,** speed patterns of nuclei (top), and corresponding NII value, all with color coding matching events in imaging sequences. Speed peaks that associate with decreasing NII values (‘phase IV peak events’) are indicated by colored dots and grey areas, and representative speed peak sizes are indicated by length of black boxes. **Right column**, speed as a function of delta NII, where values that relate to color-coded speed peaks/nuclear rounding are encircled, and numbers in green, median value of respective delta NII. Part of the image sequence from **(C)** is reproduced from [3].

**Figure S5.** **Selection and depiction scheme of phase IV speed peak/ nuclear rounding events.** **(A)** Imaging sequence and segmentation from a representative G1 phase HT1080-Fucci cell nucleus migrating in collagen (1.7 mg/ml) in the presence of GM6001. **(B)** Analysis of the nuclear parameters speed and NII, and simultaneous display over time to demonstrate how phase IV nuclear events were selected according to when a speed peak was associated with a decrease of NII from one time step to the next. Phase IV events are marked by colored dots and vertical pastel-colored stripes, and the corresponding rounded nuclear shapes in **(A)** are marked by red-colored outline. **(C)** Phase IV (pink dots) and non-phase IV (black dots) events from G1 phase cell nuclei from cells migrating in collagen (1.7 mg/mL) and in the absence and presence of GM6001, where speed is shown as a function of delta NII values per time step (data points and cell numbers correspond to figure 3*e* right;*f*). Colored ellipses indicate increased shape change of phase IV events in right graph as compared to left graph.

**Movie legends**

**Movie S1. Spontaneous migration of mesenchymal HT1080-Fucci cells over different interphase cell cycle phases.** Migrating HT1080-Fucci cells in 3D collagen in the presence of GM6001 were imaged by pathway microscopy at 37°C. Fluorescently labeled nuclei (**middle panel**) were overlaid with brightfield signal showing polarized cell bodies within 3D collagen in different layers (**left panel**). Marked cell in left-upper corner migrated to the right-lower corner while it transited from G1 (pink) to S-Start (yellow) and S/G2 (green) cell cycle phase. In cell track, segments of phase IV peak step-to-step distances, when appearing with nuclear rounding (see colored dots and arrowheads figure S2C), were marked in respective Fucci color. **Right panel,** respective crops from the nucleus of the marked cell during cell cycle transition, with forward moving tip of nucleus being directed towards the bottom, together with Otsu-segmented outline, complementary to figure 1*a* and S2A, and used for quantifications in figure S2B-D. Time as indicated. Bar, 50 μm.

**Movie S2. Phase IV peak events in migratory G1 phase HT1080 cells.** Segmentation of in focus-HT1080 cell nuclei from G1 (as well as S/G2) phase during cell migration in collagen in the presence of GM6001, cropped from overview image sequences such as Movie S1. In the first part of the movie only the nuclear morphologies are shown, whereas in the second part centroids per segmented nucleus are shown in blue, together with centroid-connecting cell tracks where ‘phase IV events’ are marked by pink (or green) track segments. G1 phase nucleus sequence marked with asterisk is displayed in figure S5A. Time as indicated.

**Movie S3. Illustration of the nuclear shape rotation method for fluctuation analysis.** The two left columns show the previous and current frames of a sequence separated by 4 minutes each. The second column to the right shows the anticlockwise rotation in 1 degree steps of the ‘current’ over the ‘previous’ segmented frame. Non-overlapping areas are shown in pink and green. The right column reflects the ‘best fit’ with maximum overlap of the 2 shapes, where 0 means complete overlap. Note that the ‘best fit’ is updated whenever a new maximum overlap is found during the rotation. This analysis tool corresponds to fluctuation analysis used in figure 1*j*.

**Movie S4. HT1080 dual-color cell in dense collagen: oscillatory migration and repeated rapid rounding of the nucleus.** Cell migrates within bovine collagen (3.3 mg/ml; reflection signal) by shape change of cell body (DsRed2) and nucleus (H2B-eGFP), as imaged by confocal microscopy at 37°C over 5 hours (left). Each computed outline of cell body and nucleus contains a centroid (blue) that progressively forms migration paths (red). Long path segments marked in cyan, green, orange and yellow color in both cell body and nucleus associate with rapid nuclear rounding (grey filling) and represent ‘phase IV peak events’. Progressing outline of cell body stalls at 135 min as pseudopod leaves image field (black arrowhead). Nucleus outlines presented either as simple progressing (middle), progressive overlay of outlines (second to the right) and as overlay of grey rounded areas after oscillatory velocity peaks. Movie corresponds to figure 2*b*. Time as indicated. Bar, 10 μm.

**Movie S5. Migration of HT1080 cells through synthetic microdevice.** Cells transfected with NLS-GFP migrated through indicated 10 μm^2^ and 75 μm^2^ pores of a microdevice over 9 h (first movie part) or around 1 h (second movie part). Green, NLS-GFP signal; transmission, PDMS pillars and cell bodies. Arrowheads indicate examples of nuclear passage through pore center, of which events were used for figure 3*c* (first movie part) or figure 2*e* (second movie part, with long phase IV-associated trajectory in cyan).

**Movie S6. TSA reduces cell migration efficacy and nuclear shape change.** HT1080 G1 phase (Fucci-red) cells, after pre-treatment by DMSO or TSA moved in collagen in the presence of GM6001. Note the size increase of the nucleus after pre-treatment by 500 ng/mL TSA. Comparison of the original fluorescent nuclear shapes (left column) with thresholded segmented counterparts (middle) and outlines (right) illustrate segmentation quality used for this study. Complementary with figure 4*e*. Time as indicated.

1. Nishitani, H., et al., *The human licensing factor for DNA replication Cdt1 accumulates in G1 and is destabilized after initiation of S-phase.* J Biol Chem, 2001. **276**(48): p. 44905-11.

2. Krause, M., J. Te Riet, and K. Wolf, *Probing the compressibility of tumor cell nuclei by combined atomic force-confocal microscopy.* Phys Biol, 2013. **10**(6): p. 065002.

3. Denais, C.M., et al., *Nuclear envelope rupture and repair during cancer cell migration.* Science, 2016. **352**(6283): p. 353-8.
